# Costing Analysis of a Pilot Community Health Worker Program in Rural Nepal

**DOI:** 10.9745/GHSP-D-19-00393

**Published:** 2020-06-30

**Authors:** Prajwol Nepal, Ryan Schwarz, David Citrin, Aradhana Thapa, Bibhav Acharya, Yubraj Acharya, Anu Aryal, Aaron Baum, Ved Bhandari, Laxman Bhatt, Dipak Bhattarai, Nandini Choudhury, Binod Dangal, Meghnath Dhimal, Santosh Kumar Dhungana, Bikash Gauchan, Scott Halliday, SP Kalaunee, Lal Bahadur Kunwar, Duncan Maru, Isha Nirola, Rashmi Paudel, Anant Raut, Hari Jung Rayamazi, Sabitri Sapkota, Dan Schwarz, Poshan Thapa, Pratistha Thapa, Aparna Tiwari, Roshani Tuitui, Eric Walter, Sheela Maru

**Affiliations:** a Possible, New York, NY, USA.; b Brigham and Women’s Hospital, Department of Medicine, Division of Global Health Equity, Boston, MA, USA.; c Harvard Medical School, Department of Medicine, Boston, MA, USA.; d Massachusetts General Hospital, Department of Medicine, Division of General Internal Medicine, Boston, MA, USA.; e University of Washington, Department of Global Health, Seattle, WA, USA.; f University of Washington, Department of Anthropology, Seattle, WA, USA.; g University of Washington, Henry M. Jackson School of International Studies, Seattle, WA, USA.; h Icahn School of Medicine at Mount Sinai, Arnhold Institute for Global Health, New York, NY, USA.; i Nyaya Health Nepal, Kathmandu, Nepal.; j University of California, San Francisco, Department of Psychiatry, San Francisco, CA, USA.; k Pennsylvania State University, College of Health and Human Development, Department of Health Policy and Administration, University Park, PA, USA.; l Nepal Health Research Council, Kathmandu, Nepal.; m University of California, San Francisco, Health Equity Action Leadership Initiative, San Francisco, CA, USA.; n Eastern University, College of Leadership and Development, St. Davids, PA, USA.; o Icahn School of Medicine at Mount Sinai, Department of Health Systems Design and Global Health, New York, NY, USA.; p Icahn School of Medicine at Mount Sinai, Department of Internal Medicine, New York, NY, USA.; q Icahn School of Medicine at Mount Sinai, Department of Pediatrics, New York, NY, USA.; r Harvard T.H. Chan School of Public Health, Boston, MA, USA.; s Beth Israel Deaconess Medical Center, Department of Medicine, Boston, MA, USA.; t Ariadne Labs, Harvard T.H. Chan School of Public Health and Brigham and Women’s Hospital, Boston, MA, USA.; u University of New South Wales, School of Public Health and Community Medicine, Sydney, NSW, Australia.; v Nursing and Social Security Division, Dept of Health Services, Kathmandu, Nepal.; w University of Pennsylvania, Perelman School of Medicine, Philadelphia, PA, USA.; x University of Pennsylvania, The Wharton School, Healthcare Management Department, Philadelphia, PA, USA.; y Icahn School of Medicine at Mount Sinai, Department of Obstetrics, Gynecology and Reproductive Science, New York, NY, USA.

## Abstract

Community health workers (CHWs) are essential to primary health care systems and are a cost-effective strategy to achieve the Sustainable Development Goals (SDGs). Nepal is strongly committed to universal health coverage and the SDGs. In 2017, the Nepal Ministry of Health and Population partnered with the nongovernmental organization Nyaya Health Nepal to pilot a program aligned with the 2018 World Health Organization guidelines for CHWs. The program includes CHWs who: (1) receive regular financial compensation; (2) meet a minimum education level; (3) are well supervised; (4) are continuously trained; (5) are integrated into local primary health care systems; (6) use mobile health tools; (7) have consistent supply chain; (8) live in the communities they serve; and (9) provide service without point-of-care user fees. The pilot model has previously demonstrated improved institutional birth rate, antenatal care completion, and postpartum contraception utilization. Here, we performed a retrospective costing analysis from July 16, 2017 to July 15, 2018, in a catchment area population of 60,000. The average per capita annual cost is US$3.05 (range: US$1.94 to US$4.70 across 24 villages) of which 74% is personnel cost. Service delivery and administrative costs and per beneficiary costs for all services are also described. To address the current discourse among Nepali policy makers at the local and federal levels, we also present 3 alternative implementation scenarios that policy makers may consider. Given the Government of Nepal’s commitment to increase health care spending (US$51.00 per capita) to 7.0% of the 2030 gross domestic product, paired with recent health care systems decentralization leading to expanded fiscal space in municipalities, this CHW program provides a feasible opportunity to make progress toward achieving universal health coverage and the health-related SDGs. This costing analysis offers insights and practical considerations for policy makers and locally elected officials for deploying a CHW cadre as a mechanism to achieve the SDG targets.

## Abstract (precis)

Data from a retrospective costing analysis offers insights and practical considerations for policy makers and locally elected officials for designing and implementing a new community health work cadre as a mechanism to achieve SDG targets in Nepal.

## Teaser

**Key Findings:**

- The average per capita annual cost of a pilot community health worker (CHW) program in rural Nepal is US$3.05.
- Personnel costs, the largest cost driver, contribute 74% of the total implementation costs and are affected by the number of households covered, population distribution, geographical terrain, and supervision structure.

**Key Implications:**

- Policy makers should consider further expansion and improvement of community health systems to make progress toward achieving universal health coverage and the health-related Sustainable Development Goals.
- Policy makers may benefit from considering alternative implementation scenarios that explore 3 challenges with CHWs: payment amount, supervision structure, and integration of new cadres into local primary health care systems.

## INTRODUCTION

As the global community works to collectively realize our commitment to universal health coverage (UHC) and the Sustainable Development Goals (SDGs), robust community health care systems will be a critical foundation.^1^ Community health worker (CHW) programs have increasingly received attention and focus as a key strategy to achieve the SDGs and UHC, with dramatic increases in global investments and scale-up of national and subnational programs.^2^ Strong evidence suggests the cost-effectiveness of CHW programs, with economic returns of up to 10:1.^3^ The World Health Organization (WHO) has recently endorsed them as a key mechanism to achieve UHC and SDG 3—ensure healthy lives and promote well-being for all at all ages—in the first global guidelines for CHW program design and implementation,^4^ offering important guidance to policy makers and locally elected officials looking to improve progress toward SDG targets.

Nepal has made important gains in health outcomes, including a two-thirds decline in maternal mortality and halving rates of stunting between 1990 and 2015.^5^ Despite this, similar to many countries, Nepal is not presently on track to meet its SDG targets by 2030,^6^^,^^7^ with maternal mortality at 239 per 100,000 live births, under-5 mortality at 39 per 1000 live births, and 38% of disability associated with noncommunicable diseases (NCDs) occurring before age 40 years.

### Community Health Care in Nepal

For decades, Nepal has been a leader in community health care systems. The country has a long history of various CHW models, including both full-time and part-time and paid and voluntary cadres, covering a range of programmatic outreach and service delivery foci.

In the 1980s, the Ministry of Health introduced full-time, paid village health workers (VHWs), who received 6 weeks of primary health care training and focused mainly on increasing immunization. After the VHW program was well established, community health leaders were added to support the VHWs, promote the health messages they were spreading, and increase community participation in improving the nation’s health.^8^ Unlike VHWs, community health leaders were unpaid volunteers who received only 1 month of training and did not make home visits, but rather coordinated convenient places and time for people to meet them elsewhere in the community.^8^

In 1988, the government initiated the voluntary, part-time cadre of female community health volunteers (FCHVs) to focus on increasing uptake of family planning methods along with immunizations. Both of these groups of health care workers were part of Nepal’s response to the global movement toward primary health care that emerged out of the 1978 Alma Ata Declaration.^9^ The FCHV program was developed as a country-level response to the global focus on primary health care and has grown to more than 50,000 volunteers nationally. The FCHV program has been a pillar of improved health care outcomes in Nepal, including national priorities of vitamin A distribution, immunization, and antenatal and postnatal care.^10^ In 2010, Nepal was recognized as a leading country in progress toward achieving the Millennium Development Goals, and the FCHV program was acknowledged as a key component in that effort.^11^

In the early 1990s, in line with the new National Health Policy of 1991 aimed at bringing health care services closer to rural communities, the government introduced the maternal and child health worker (MCHW).^12^ Also a full-time, paid position, MCHWs were charged alongside FCHVs with covering entire village development committees, the smallest geo-administrative unit at the time, and were affiliated to rural health care facilities such as primary health care centers and subhealth posts. As educational levels and training opportunities increased over time,^13^ VHWs and MCHWs were transitioned to auxiliary nurse-midwives (ANMs) and auxiliary health workers (AHWs), respectively, both of which undergo 18 months of pre-service training. In parallel, the FCHV program continued to grow and now includes more than 50,000 volunteers covering communities throughout all of Nepal’s 7 provinces—roughly 1 FCHV per every 500–1000 people in the country, and their areas of responsibility have broadened to include a range of other maternal, neonatal, and child health outreach and services.^12^

Despite Nepal’s significant successes in community health, in particular the FCHV program, there are areas for improvement for community-based cadres. These include having more robust managerial and training structures, establishing minimum educational requirements, expanding to full-time paid employment (FCHVs are part-time volunteers working on average 7.2 hours per week), and addressing supply chain management challenges similar to much of the rest of the health care system.^14^^–^^16^ Although improvements are being made to the FCHV program, including in the new *FCHV Strategy 2076* that will outline a requirement for new FCHVs to have a minimum educational requirement, as well as other efforts at local levels where FCHVs are advocating for improved structure, supervision, and payment, these challenges make it unlikely that the FCHV program in its current state presents a viable pathway toward the SDGs. Indeed, in these regards, increasingly over the last decade, policy discussions and pilot programs have examined leveraging additional community-based cadres, including ANMs, to expand community health.^17^

Nepal recently transitioned to a federal republic, including further decentralization of the health care system. Newly elected municipal governments are seeking locally appropriate strategies to better respond to their constituents’ health care priorities. Although this transition and decentralization process has brought significant challenges, paired with Nepal’s commitment to UHC and the SDGs, the new political context is also an excellent foundation for improved municipality-based CHW service delivery.

### Improving Community Health Care Models in Nepal

To meet the gap in SDG targets in Nepal, it has become clear that new ideas and investments need to be made in CHW systems as part of overall health care systems strengthening efforts.^14^^,^^15^^,^^18^^,^^19^ The WHO guidelines,^4^ and other recent recommendations^2^^,^^20^ for design of effective CHW programs, offer helpful framing in these regards, highlighting that CHWs should: (1) receive regular financial compensation; (2) meet a minimum education level; (3) be well supervised; (4) be continuously trained; (5) be closely integrated into the local primary health care system; (6) use a mobile health tool; (7) have consistent supply chain; and (8) live in the communities they serve.

**FIGURE 1.**
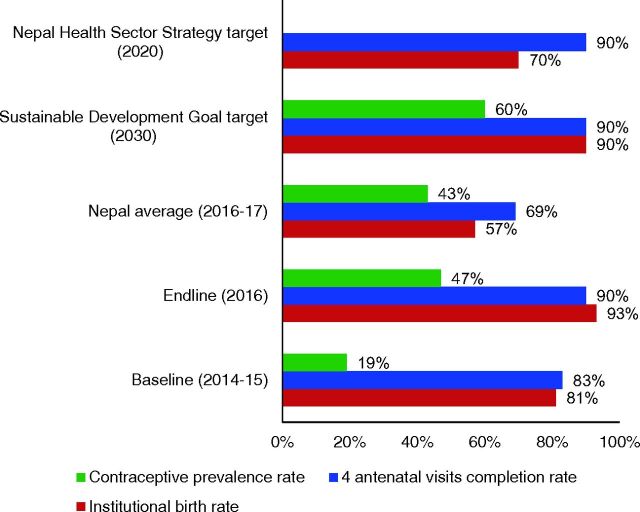
Preliminary Health Care Outcomes of Pilot CHW Program in Nepal, Compared to National and Global Targets^a^ Abbreviations: CHW, community health worker. ^a^ Data published previously^22^ and reproduced here in parallel to Sustainable Development Goal and Nepal Health Sector Strategy targets.

In this context, the nongovernmental organization Nyaya Health Nepal developed a collaborative effort with the Ministry of Health and Population, Department of Health Services, Family Welfare Division to implement a CHW pilot program^21^ that was closely aligned with WHO guidelines (Table 1), which may offer insight for Nepal’s future CHW programs. This pilot program has had promising early results, described in detail elsewhere,^22^^,^^23^ including between 2014 and 2016, improving antenatal care (ANC), institutional birth rate, and postpartum contraception (Figure 1). Since 2016, monitoring data collected in the course of program operations has shown a further increase in the institutional birth rate in the catchment area to 96% (unpublished data). More recently, in multiple municipalities across the country, locally elected officials are planning or have already begun implementing new community-based services, including adjustments to the FCHV program as well as introduction of new cadres with a range of training and skillsets (e.g., ANMs). Despite growing interest, there is limited consensus or coordination of how such programs or new cadres should be implemented.

**TABLE 1.** Pilot CHW Program Design in Relation to WHO Guidelines

**WHO Recommended CHWProgram Attributes** ^4^	**CHW Pilot Program Descriptive Characteristics**	**Program Alignment WithWHO Recommendations**
Selection	CHWs: Have minimum education requirement of 10th level Locally selected women who live in the community they serve	✓
Training	Includes both preservice and continuous training Training combines theory and practice based in community	✓
Certification	Formal program certification is currently in process, pending government approval	–
Supportive supervision	Supervisors conduct: Regular in-person supervision including observation in community Dedicated supervision, data-driven feedback, and performance review	✓
Incentives	CHWs are salaried, full-time employees, at a rate competitive to local market conditions, paired with additional non-financial incentives	✓
Career ladder	Pending further government certification/approval	–
CHW: population ratio	Ratio is based upon epidemiology, geography, topography, security, andworkload/expected responsibilities	✓
Data collection/utilization	CHWs collect data via mHealth platform, use in monitoring and review with supervisors, use in service delivery improvements	✓
Community integration	CHWs mobilize resources and promote health and social needs	✓
Supply chain	CHWs have reliable and consistent supply of medicines and supplies closely linked to local primary health centers	✓

Abbreviations: CHW, community health worker; WHO, World Health Organization.

Although CHW programs may be cost-effective strategies for health care systems strengthening,^3^^,^^4^ there are limited data in Nepal regarding the costs or operational details of what a CHW program aligned with WHO guidelines would entail. Understanding what options are available as policy makers and locally elected officials consider more robust community-based service delivery will be critical to achieving SDG targets. In these regards, policy makers at federal and local levels are grappling with multiple questions, including:

- Should CHWs be paid and, if so, how much?
- How should effective supervision structures be implemented?
- How can a new CHW cadre be integrated with local primary health care systems?

Understanding the available options as policy makers and locally elected officials consider more robust community-based service delivery will be critical to achieving SDG targets.

Here, we describe costs for a catchment area population of 60,000 community members in the pilot program, including analysis of per-capita costs, service delivery costs, and administrative costs. Secondly, to situate these costs in the context of current discussions at the municipal level, we provide 3 additional implementation scenarios for the pilot CHW cadre to reflect local policy makers’ considerations. This analysis may be instructive for locally elected officials and future community health care systems policy in Nepal, and more broadly, in similar settings globally that leverage community health care strategies to achieve UHC and SDG targets.

## METHODS

### Population and Setting

In 2014, Nyaya Health Nepal began CHW program development and implementation in Achham district in Province 7 working with a catchment area population of approximately 36,000. In 2017, the pilot study was implemented in both Achham and Dolakha districts (Province 3) targeting expansion to a catchment area population ultimately of more than 250,000 people.^21^ The subpopulation of the pilot described in this analysis included the original program area, Sanfebagar municipality, and the first expansion area, Kamalbazaar municipality in Achham, which together have a population of 60,504 persons.^24^ A total of 30 CHWs were employed in the 2 pilot municipalities during the time period of July 16, 2017 to July 15, 2018.

Achham is historically one of the most impoverished districts with some of the lowest-performing health indices in the country.^5^^,^^10^ The government-owned Bayalpata Hospital in Achham is operated by a public-private partnership between the Ministry of Health and Population and Nyaya Health Nepal. Through this public-private partnership, the hospital is owned by the Ministry of Health and Population and is financed through federal, provincial, and municipal budgetary allocations, supplemented by Nyaya Health Nepal’s own financing. Nyaya Health Nepal oversees day-to-day management and operation of the facility and all health care services and is accountable to the Ministry for regular reporting via the local District Health Office. Bayalpata Hospital serves as a referral facility for comprehensive emergency obstetric and newborn services and noncommunicable disease (NCD) management, in addition to providing adult and pediatric medicine and basic surgical services for Sanfebagar. Similarly, the Achham district hospital in Mangalsen serves as the referral facility for Kamalbazaar and provides a similar range of services. Kamalbazaar also has a government primary health center that provides basic outpatient care; maternal and child health services, such as immunization, nutrition, pneumonia, and diarrhea care; pre- and postnatal care; and maternal and newborn care, including emergency obstetric services, as well as several village health posts, which provide a smaller set of basic primary and ANC services.

### CHW Program Design, Structure, and Service Delivery

For the current pilot, the initial program protocols were adapted in collaboration with local government partners and the Ministry of Health and Population, Department of Health Services, Family Welfare Division.

**FIGURE 2.**
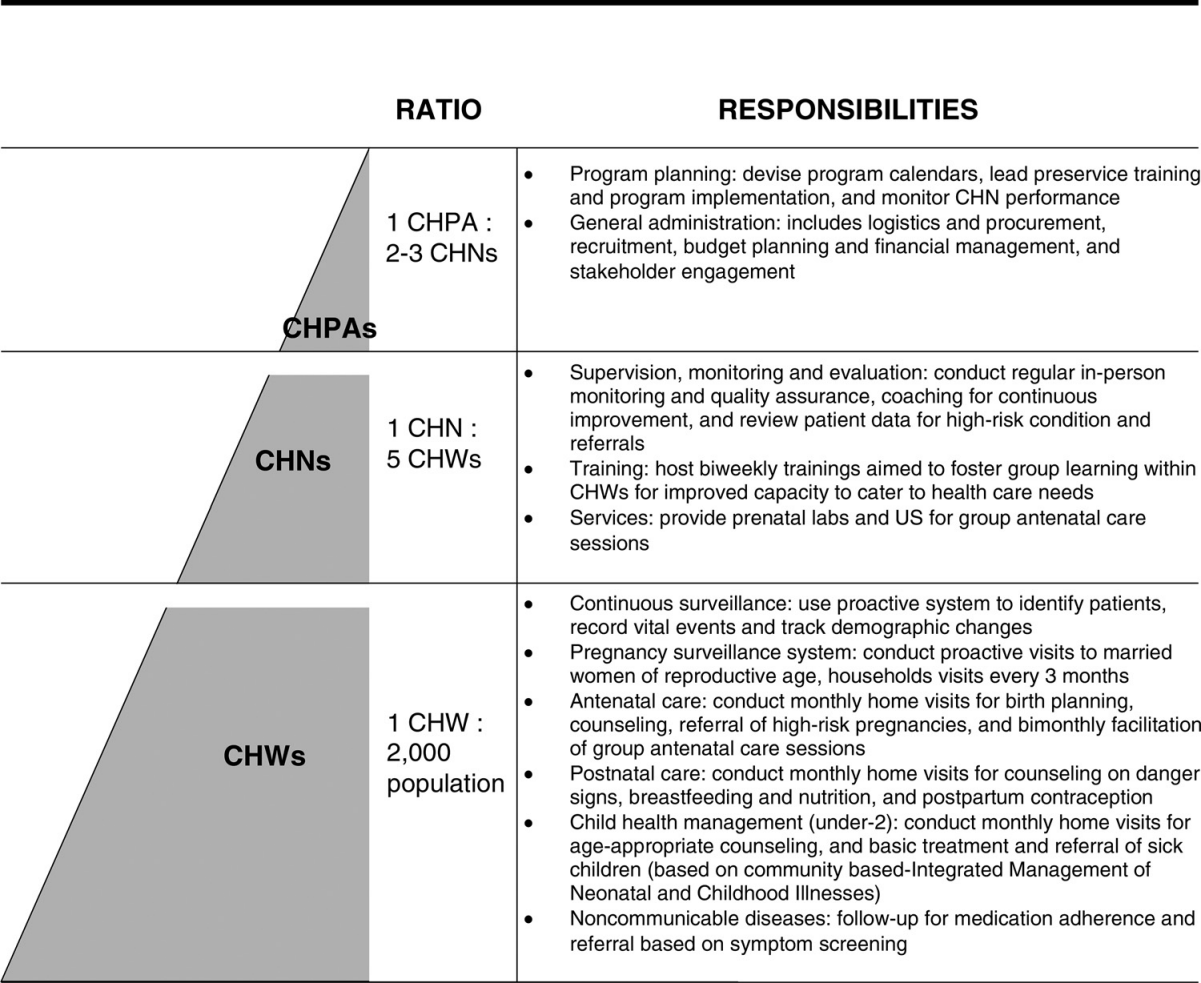
Program Design of Nepal Ministry of Health and Population, Family Welfare Division and Nyaya Health Nepal CHW Pilot Program Abbreviations: CHPA, community health program associate; CHN, community health nurse; CHW, community health worker.

All CHWs in the pilot: (1) receive regular financial compensation; (2) meet a minimum education level requirement of secondary schooling; (3) are well supervised; (4) receive continuous training; (5) are closely integrated into the local primary health care system; (6) use a mobile health tool; (7) have a consistent supply chain; (8) live in the communities they serve; and (9) provide service without point-of-care user fees. CHWs are full-time, paid employees of Nyaya Health Nepal and assigned to individual villages, or “wards,” according to the political designation of the new federal system. CHW are paid a starting base salary of Nepalese rupees (NPR) 19,930 per month. Nyaya Health Nepal’s procurement and operations teams provides CHWs with supplies to use in their daily work, such as mobile phones, urine pregnancy tests, blood pressure cuff, measuring upper arm circumference tape, and other basic supplies. Each CHW is responsible for a population of, on average, 2,000 residents. In a few areas, where the population is very spread out and the terrain is more challenging, an additional CHW is employed to cover the ward. All CHWs have dedicated supervision, with 1 community health nurse (CHN) supervising, training, and conducting monitoring and evaluation for 5 CHWs. Community health program associates supervise 2–3 CHNs and are responsible for program planning and administration (Figure 2). CHWs receive 1 month of preservice training, followed by 2-week training modules each month over the following 3 months after baseline family registration is complete. Continuous ongoing trainings are conducted at monthly CHW meetings.

CHWs are engaged with several subpopulations within their catchment area: married women of reproductive age, children aged 2 years and younger, pregnant and postpartum women, and adults with chronic diseases. For each of these subpopulations, the CHWs maintain a regular home visit schedule and conduct counseling, case detection, and referrals. During home visits, CHWs conduct urine pregnancy testing; trimester-specific antenatal counseling and referral; postnatal counseling and referral (with a strong emphasis on contraception counseling); age-specific child health and nutrition counseling; childhood illness screening and referral (based on community-based Integrated Management of Neonatal and Childhood Illnesses, and NCD counseling and referral (Figure 2).^21^ Services delivered are determined based upon local and national priorities and morbidity and mortality burden. In addition to home visits, CHWs facilitate group ANC sessions at local health posts on a bimonthly schedule, with 1 of the monthly visits for early gestation (4- and 6-month pregnant women) and the other for late gestation (8- and 9-month pregnant women).

CHWs coordinate with the existing primary health care system (including local village clinics, primary health care centers, and hospitals) through monthly data sharing and following up on referrals made. They also join monthly FCHV meetings at local village clinics and coordinate with FCHVs to ensure that all pregnant women and children are identified and receiving care. The program does not alter public health services coordinated and delivered in the study areas through the Government of Nepal with the exception of group ANC. The CHWs work very closely with government ANMs at village health posts to facilitate group ANC sessions. These sessions are intended to replace individual ANC visits, as physical assessments and medication distribution are conducted during the group along with discussion and counseling. Sometimes women present outside of group visits for individual ANC, but group ANC sessions fulfill all basic ANC requirements. Additionally, CHNs provide antenatal ultrasound and prenatal lab services (including hemoglobin, HIV, blood typing, hepatitis B and C, and urine glucose and protein) during these sessions.^25^^,^^26^ CHNs receive preservice training as well as ongoing training and assessment to assure the quality of these services. The role of FCHVs is not altered in the study areas.

CHWs are equipped with Android-based smartphones utilizing the CommCare platform^27^ for clinical service documentation and content to support counseling and referrals. The data from the CHW smartphones are shared with the Nyaya Health Nepal hospital facility-based electronic health records to aid in providing continuity of care between community and facility-based services.^23^ The supply chain is managed through a digital inventory management system linked to Nyaya Health Nepal’s facility-based electronic health record system. No user fees are charged for services delivered by CHWs. The structure is closely aligned with WHO guidelines for the design and implementation of CHW programs (Table 1).^4^^,^^23^

The pilot study has been described in detail previously.^21^ In brief, the pilot is a Type II hybrid effectiveness-implementation research study, evaluating effectiveness using a pre-post, quasi-experimental design with stepped implementation and evaluating implementation using the RE-AIM framework. Enrollment began in 2017 and concluded in 2019, with the goal to enroll over 250,000 community members across both Dolakha and Achham districts. The analysis included in this discussion focuses on a catchment area population only in Achham district of 60,504 persons across 2 municipalities (Table 2): Sanfebagar municipality (population 36,766) and Kamalbazaar municipality (population 23,738). The pilot is ongoing and future analysis will include data for the full catchment area.

**TABLE 2.** Description and Summary Statistics from Catchment Area of CHW Pilot Program, Including Sanfebagar and Kamalbazaar Municipalities

	**Sanfebagar**	**Kamalbazaar**	**Total**
Total population	36,766	23,738	60,504
Direct service beneficiaries	10,816	8,222	19,038
CHW: population ratio	1:1,935	1:2,158	1:2,017
Community health nurses: CHW ratio	1:4.8	1:5.5	1:5
Total costs (USD$)	118,327	66,177	184,504
Number of CHWs	19	11	30
Number of CHNs	4	2	6
Number of CHPAs	2	1	3
Cost per capita (USD$)	3.22	2.79	3.05

Abbreviations: CHN, community health nurse; CHPA, community health program associates; CHW, community health worker.

### Costing Analysis

We performed a retrospective costing analysis using demographic, programmatic, and financial data for the period between July 16, 2017, and July 15, 2018. All programmatic information — including number of households enrolled in the CHW program, number of beneficiaries receiving care, CHW encounters and service delivery, and noncare-delivery events, such as trainings and supervision field visits, were collected from the CommCare platform and staff program calendars. CHWs use CommCare to collect household visit data, and these data formed the basis for analyzing CHW resource utilization during care delivery.^23^ All direct costs (personnel, medicines and consumables, and depreciation of equipment) and indirect costs (transportation, regular supplies, staff benefits, and depreciation of digital tools) were obtained from organizational financial records.

The Nepal Health Research Council (#461/2016) and Brigham and Women’s Hospital Institutional Review Board (2017P000709/PHS) approved the study. Verbal informed consent was obtained by CHWs for delivery of patient care and for use of a limited identifiable dataset for research analysis.

We employed a ‘top-down’ costing methodology based upon methods previously described by the Joint Learning Network.^28^ This methodology first disaggregates all direct and indirect costs into ‘intermediate cost centers’ and second into ‘final cost centers.’ Intermediate cost centers consisted of 6 care delivery programs: pregnancy case detection; individual ANC; group ANC; postnatal care; childhood illness management for children aged 2 years and younger; and NCD management. Intermediate cost centers also included 5 administrative functions: planning and administration; training; supervision, monitoring and evaluation; data reporting and learning; and, continuous surveillance. Final cost centers consisted of only the 6 care delivery programs.

To allocate costs to intermediate cost centers, we defined a ‘capacity cost rate’ for CHWs, equal to the time spent in each service encounter (available from the CommCare database) divided by the number of available minutes during the year. Personnel costs of supervisory and administrative staff (community health program associates and CHNs, as illustrated in Figure 2) were allocated to administrative functions based on resource attribution gathered from staff program calendars. Staff benefits and expenses, such as uniforms, food, and telecom network expenses, were allocated using the same methodology. Notably, costs for regional and national administrative staff were not included given these roles are primarily responsible for program design and organizational strategy, and are not anticipated to be necessary if the program scales beyond the pilot study. Similarly, initial programmatic development costs were not included. All remaining direct and indirect costs were attributed to either programs or administrative functions based on their relative use (e.g., transportation costs were partly allocated to supervisory systems and to the group ANC program, and mobile phones and associated technology support was allocated to data reporting). In summary, the allocated costs represent annual recurring costs of operating the CHW program and do not include costs relating to initial training and program development.

This approach ensured that all direct and indirect costs were allocated to the intermediate cost centers of the 6 service delivery programs or the 5 administrative functions. To arrive at final costs, all administrative function costs were further allocated downward to service delivery programs based on the CHW encounters using a step-down costing methodology.^28^ The final cost centers are the 6 service delivery programs.

We performed an analysis of programmatic cost per capita by village—the smallest geopolitical division—for which there are 14 villages in Sanfebagar municipality and 10 villages in Kamalbazaar municipality. Analysis of cost by service delivery—pregnancy case detection, individual ANC, group ANC, postnatal care, under-2 care, and NCD care—was performed for both cost per capita (of the total catchment area) and for cost per beneficiary (for persons who received the service). Notably, group ANC and NCD services were available to only 56% and 61% of the population, respectively, during the measurement period. Group ANC limitations were due to certain villages in both municipalities not having yet implemented services, and NCD services were not implemented in Kamalbazaar due to constraints at the local primary health care center for NCD management.

All costs were measured in NPRs and converted to US dollars using a conversion rate of NPR104.4 to US$1, the average exchange rate for the 1-year measurement period.^29^ Costs in NPRs are available upon request. Further costing methodology details are included in Supplement 1.

### Alternative CHW Implementation Scenarios

We performed the above-described costing analysis to generate insights regarding cost of the pilot program as policy makers consider new community-based strategies. However, in our ongoing relationships with federal and local policy makers, we recognize locally elected officials throughout Nepal are pursuing a variety of community-based implementation strategies in the new federal health context.

We performed the costing analysis to generate insights regarding cost of the pilot program as policy makers consider new community-based strategies.

To address policy makers’ current considerations regarding implementation of new community-based services, we present 3 alternative scenarios for potential implement strategies for a CHW cadre similar to the pilot program:

- Scenario 1 projects the implementation costs if CHW salaries were decreased. As there is no similar CHW cadre in Nepal currently, we used Nepal’s Labor Act^30^ definition of minimum wage to inform an ‘adjusted salary’ for CHWs at scale, determined to be US$1,829.36 annually (NPR190,985, including government benefits and pension).
- Scenario 2 projects implementation wherein administrative functions of the program are absorbed into municipal health care unit governance structures. Specifically, this scenario includes the functions performed by community health program associates (Figure 2), such as program planning, budgeting, evaluation, human resources, and financial management, to be taken on by local governance structures, thereby eliminating the community health program associate cadre.
- Scenario 3 incorporates the CHW program directly into existing primary health care infrastructure (e.g., primary health centers or health posts). In this scenario, the functions of CHNs (Figure 2) – supervision, training, and monitoring and evaluation – would be performed by government health care facility staff (e.g., ANMs, thereby eliminating the CHN cadre). Additionally, as group ANC services in this pilot are delivered in large part by CHNs, this service would be subsumed into local health care facility service delivery or discontinued.

We developed these scenarios in response to the authors’ conversations with multiple stakeholders at federal and municipal-levels highlighting 3 broad challenges: amount of payment for CHWs; CHW supervision structure; and integration of new CHW cadres into local primary health care systems. These scenarios are intended to complement the costing analysis results and offer insight into human resources and implementation possibilities as locally elected officials and federal policy makers consider the development of similar programs throughout the country.

Scenarios have not been tested but are presented for the purposes of considering alternative policy options. As each scenario also impacts cost, changes are presented in a cumulative manner in decreasing order of overall cost; the conditions and cost reductions of scenario 1 are included in scenario 2, and conditions from both scenarios 1 and 2 are included in scenario 3.

## RESULTS

### Program Cost

We analyzed the cost for pilot implementation for villages in 2 municipalities (Table 2) across multiple factors: cost per capita, by administrative function, by service delivery, cost per beneficiary, and by indirect and direct costs.

**FIGURE 3.**
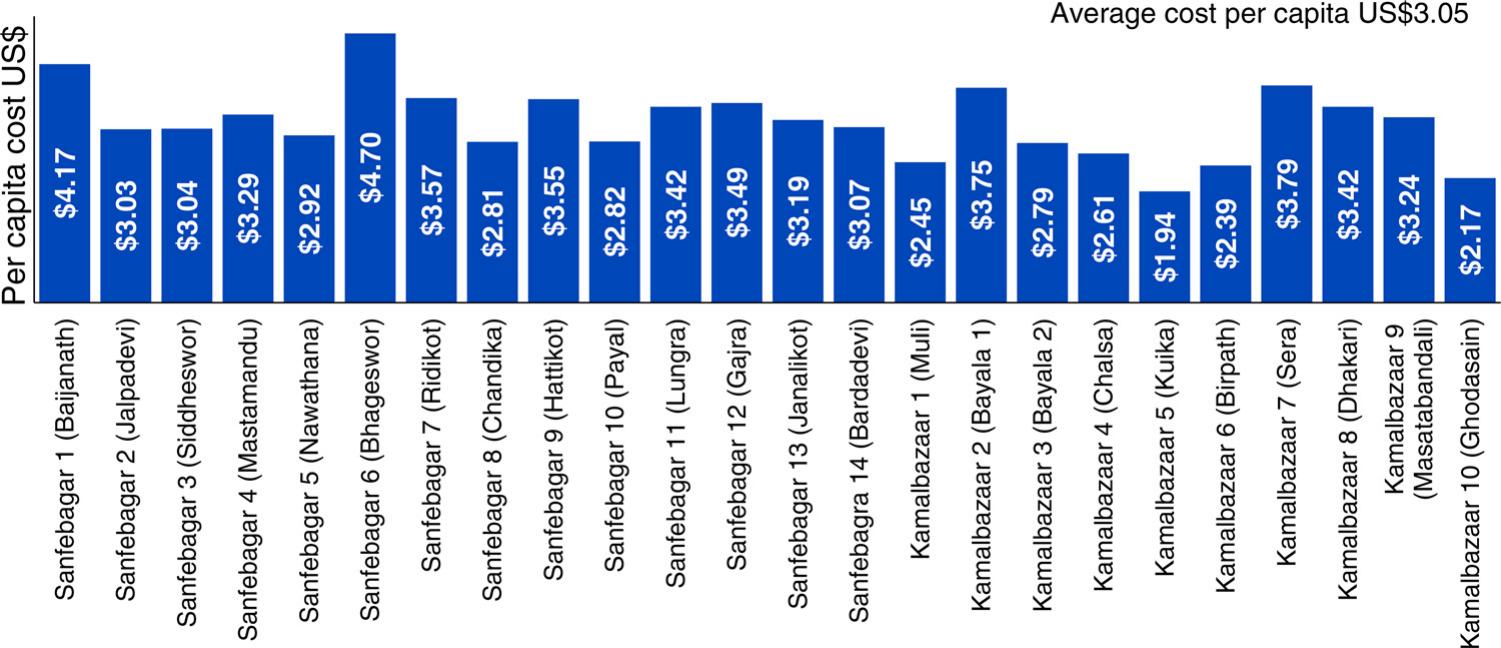
Cost Per Capita of the CHW Pilot Program in Nepal, by Village, N=24 Abbreviations: CHW, community health worker.

The analysis by village demonstrated an average cost per capita in the Sanfebagar municipality of US$3.22 and in the Kamalbazaar municipality of US$2.79 (Figure 3). The overall annual cost of the pilot CHW program during the period was US$184,504. The population-weighted average annual cost per capita across both municipalities was US$3.05. The overall cost function and variation between villages are largely driven by CHW personnel costs that tend to be a step function and increase nonlinearly at population intervals.

**FIGURE 4.**
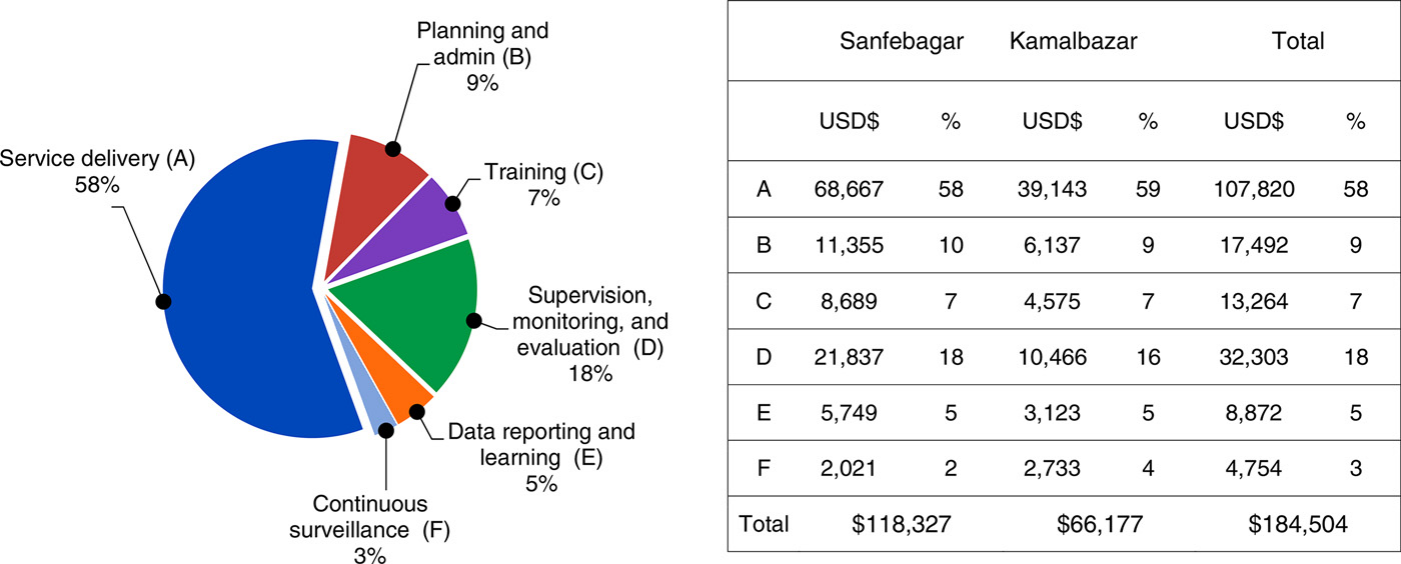
Distribution of Intermediate Cost Centers, Including Service Delivery and Administrative Functions, of Pilot CHW Program in Nepal, by Municipality Abbreviations: CHW, community health worker.

The analysis of costs by administrative function—planning and administrative; training; monitoring and evaluation, and supervision; data reporting and learning; and continuous surveillance—demonstrated that administrative functions comprised 42% of overall costs (with service delivery comprising 58%). These costs were intermediate costs and were ultimately further allocated to the 6 service delivery areas as final cost centers. The largest cost drivers in administrative functions were supervision and monitoring and evaluation, which combined comprised 18% of overall costs (Figure 4). There was no significant variation in composition of intermediate costs between Sanfebagar and Kamalbazaar municipalities.

**FIGURE 5.**
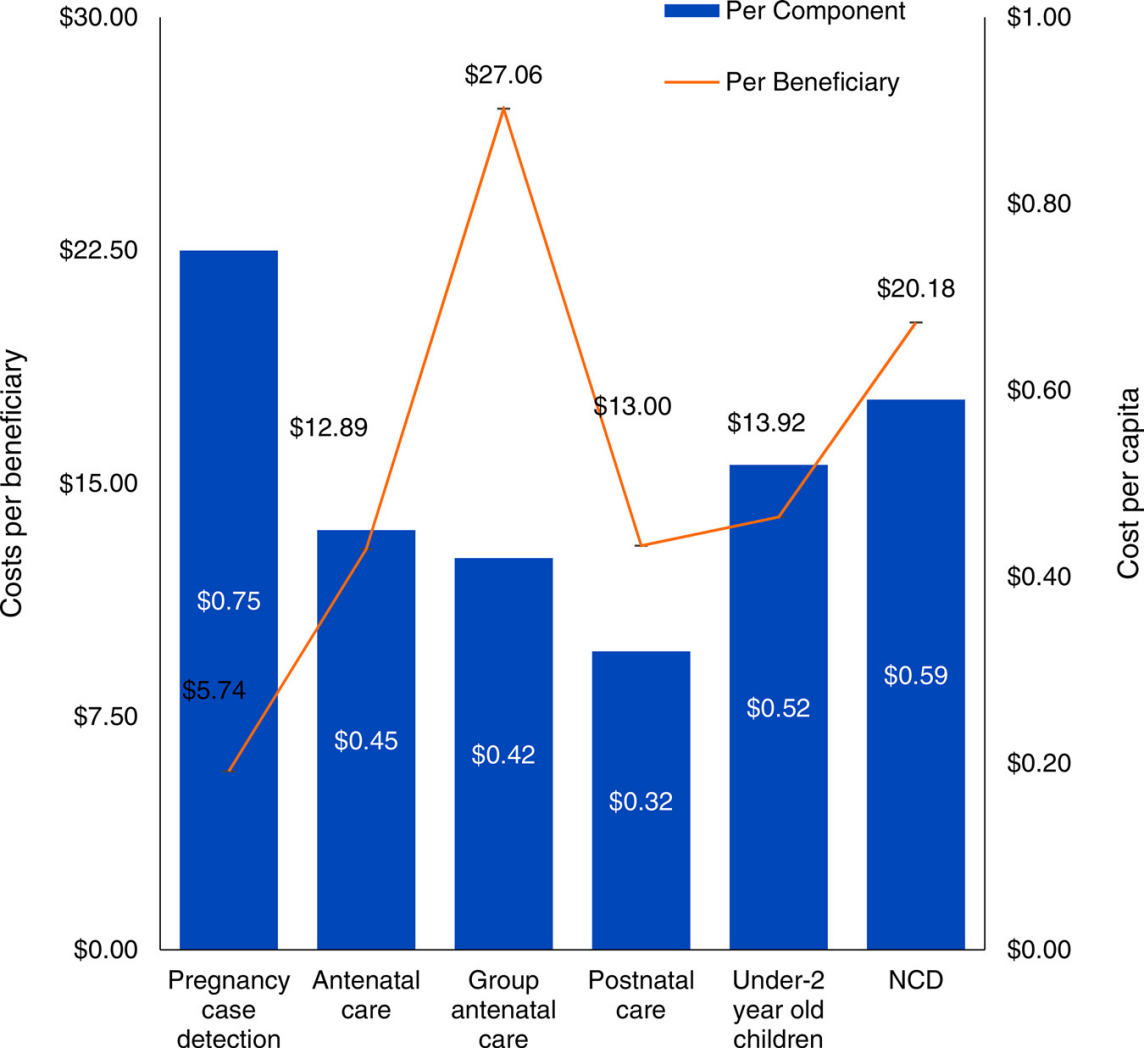
Costs Per Capita and Per Beneficiary by Service Delivery of CHW Pilot Program in Nepal Abbreviations: CHW, community health worker; NCD, noncommunicable diseases.

Analysis of final cost centers (pregnancy case detection, ANC, group ANC, postnatal care, under-2 care, and NCD) are shown in Figure 5. Pregnancy case detection was a leading overall cost driver including a per capita cost of US$0.75, and with the lowest per beneficiary cost of US$5.74. The highest cost of service per beneficiary was group ANC at US$27.06. The higher costs of group ANC were due to larger time allocation of CHNs in supervision, lower number of beneficiaries relative to home visits, and lab and diagnostic services provided during group sessions.

**FIGURE 6.**
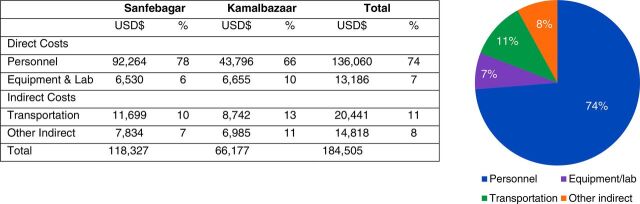
Costs by Functional Type of Expenditure in a CHW Pilot Program in Nepal Abbreviations: CHW, community health worker.

Analysis of cost by functional type of expenditure (direct costs covering personnel, equipment and consumables, and indirect costs disaggregated into transportation and other) demonstrated direct costs constitute 81% of overall costs, including staff costs of 74%, and transportation the second leading cost driver at 11% (Figure 6). In comparing municipalities, personnel in Sanfebagar comprise 78% of costs but only 66% of costs in Kamalbazaar. This is likely due to the fact that CHW-to-population ratio and CHN-to-CHW ratios were lower in Kamalbazaar than in Sanfebagar (Table 2) largely due to geopolitical village boundaries as CHWs are hired by village. Full program costing is included in Supplement 2.

### Costs of Alternative Implementation Scenarios

**FIGURE 7.**
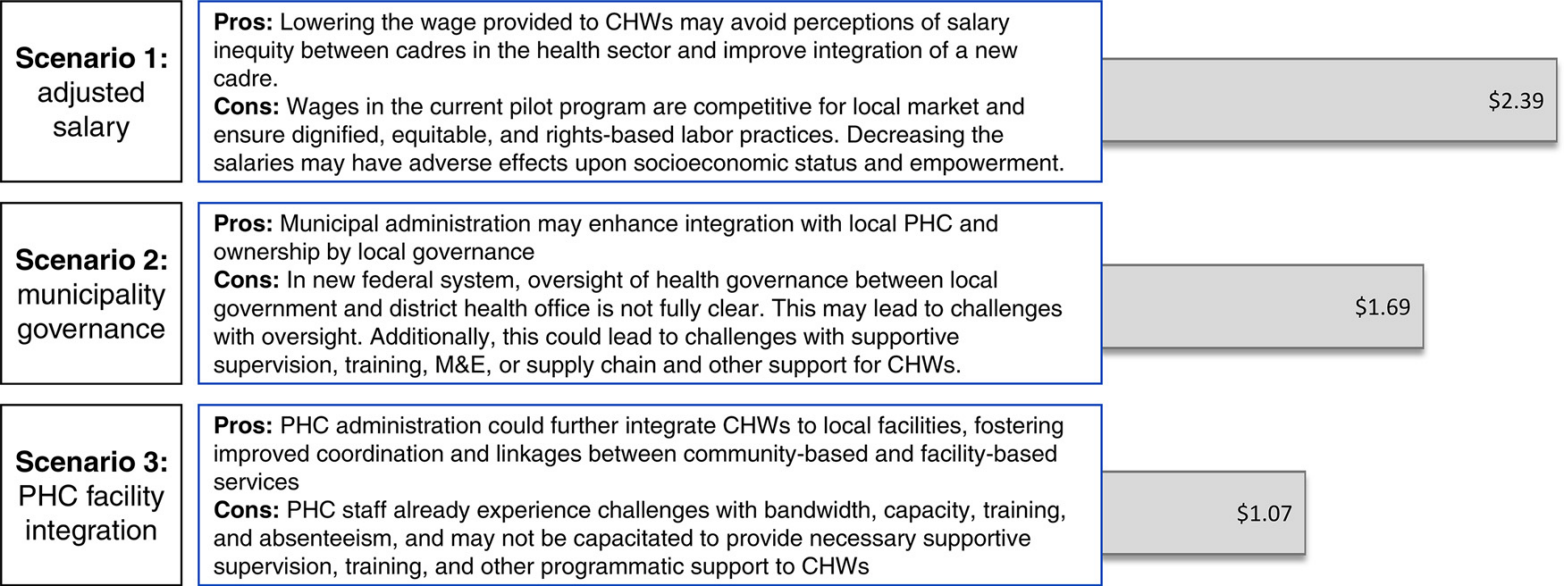
Potential Advantages and Disadvantages and Additional Per Capita Cost of 3 Alternative CHW Program Implementation Scenarios, US$ Abbreviations: CHW, community health worker, PHC, primary health care.

The projected additional per capita costs over and above current public-sector budgetary allocation for the 3 alternative implementation scenarios of a CHW cadre are shown in Figure 7. In the first scenario, CHWs would receive an ‘adjusted salary’ of US$1,829.36 annually, as per minimum wage stipulations in the Nepal Labor Act,^30^ which would decrease overall program costs by 21% from US$3.05 to a per capita cost of US$2.39. For scenarios 2 and 3, while additional per capita costs decrease with integration into municipal governance bodies and primary health care facilities, public-sector staffing would be required to absorb some of the administrative costs of CHW program oversight. These costs would be contingent upon the implementation strategy and are not accounted for in the per capita cost presented. The second scenario wherein program administration is by municipal health care units would decrease costs by 30% for an additional per capita cost of US$1.69. The third scenario would integrate CHWs into local primary health care facilities, decreasing costs by 36%, for an additional per capita cost of US$1.07.

While these scenarios have not been tested in the current pilot, Figure 7 also describes potential advantages and disadvantages for each. Cost per capita and per beneficiary for each scenario and further details of costing are included in Supplement 2.

## DISCUSSION

We describe the costs of a pilot CHW cadre, aligned with WHO’s guidelines for CHW program design, to provide operational and financial insight to policy makers considering new community-based services. The costs for the program were, on average, US$3.05 per capita, with variation per service delivered (Figure 5). Similar to other community health care programs, costs were largely due to human resources and transportation (Figure 6).^31^^,^^32^

Program costs were an average US$3.05 per capita and were largely due to human resources and transportation.

Pregnancy case detection and NCD services included some of the higher costs per capita, though pregnancy case detection had the lowest cost per beneficiary. Pregnancy case detection was the largest program component by beneficiaries count in both municipalities, whereas NCD services was the second largest in the Sanfebagar area. Group ANC services comprised the highest per beneficiary cost due to inclusion of laboratory tests and ultrasound services provided during sessions and presence of CHNs at all sessions (with corresponding increased transportation costs). Although women in areas where group ANC was not offered were also encouraged to receive these diagnostic tests, services occurred at higher-level health care facilities where available and thus were not included in the cost of CHW program delivery. Costs in the Sanfebagar municipality were higher, on average, than in Kamalbazaar due in large part to a higher staffing to population ratio (Table 2). We did not include a cost-benefit analysis as a part of this study due to limitations in data availability.

Nepal, like many countries, has a strong high-level commitment to UHC and the SDGs. A CHW cadre, such as the pilot described, offers one potential path forward for Nepal and other countries. Although it is difficult to compare costs across programs or geographies and our current analysis is not a cost-effectiveness analysis, the costs of the program described are broadly aligned with, if not perhaps cheaper than, programmatic costs in other community-based programs.^3^^,^^31^^,^^33^^–^^35^ Current health care spending in Nepal is US$51 per capita, or 6.7% of the 2016 gross domestic product (GDP), and Nepal’s SDG targets have 2 important implications for health care delivery. First, to reach the SDG goals, Nepal intends to increase per capita health care spending from US$51 to US$175 by 2030. The majority of this growth will come from Nepal’s intended increase in GDP per capita from US$759 to US$2,500 by 2030, while health care contributions will improve only marginally, from 6.7% to 7.0% of GDP. Second, to expand financial risk protection for citizens, Nepal intends to reduce the share of out-of-pocket spending from 52% of total health care expenditure (2016) to 35% by 2030. Accordingly, public-sector contributions to health care will need to increase by 12% annually in real terms, from US$13.60 of government-funded per capita health care spending (2016) to US$77 per capita by 2030. Further details are included in Supplement 3. As the Government of Nepal increases the fiscal space dedicated to health care, it will need to further incorporate CHWs as part of overall health care systems strengthening efforts.^36^

The recently established federal system of governance, decentralized health care administration, and the newly elected municipal governments throughout Nepal provide an important opportunity for enhanced community health delivery. The costs presented here provide insight into what is required to deploy a CHW cadre closely aligned with WHO guidelines. Conversations regarding implementation of new community-based cadres are already occurring at the federal and municipality levels where newly elected officials are eager to improve health indices for their constituencies. Although the costs of CHW service delivery are a concern to policy makers, with increased health care spending, including if Nepal spends the recommended 7.0% of 2030 GDP on health care required to meet its SDG targets, the allocation required for a cadre as described in the pilot may be quite feasible. Having said that, there are multiple other concurrent demands on the MOHP budget that would compete for additional funding were it to materialize.

The 3 alternative implementation scenarios presented provide additional insight for policy makers and locally elected officials. Further research is required to draw conclusions regarding impact and cost, but the scenarios reflect ongoing conversations at both federal and local municipal levels presently. As highlighted in Figure 7, there are advantages and disadvantages that must be accounted for in considering policy approaches to deploy CHW cadres. In these regards, we caution policy makers from accounting only for budgetary implications, as overall programmatic effectiveness may suffer with more limited supervision and administrative oversight (e.g., scenarios 2 and 3), thereby potentially negating the investment and limiting progress toward UHC and health-related SDG targets. Growing evidence globally demonstrates the importance of strong supervision, which should be taken into account as implementation strategies are considered.^4^^,^^37^^–^^39^ Additionally, although scenarios 2 and 3 require less per capita budgetary allocation over and above the current health care budget, they also require public-sector staffing adjustments to ensure adequate CHW programmatic oversight, and these costs have not been accounted for. Thus, we encourage policy makers to consider most importantly how implementation of a CHW cadre can be locally owned with significant community and local governance engagement, be well integrated into existing primary health care systems, and have the necessary supportive supervision to optimize impact.

We encourage policy makers to consider how implementing a CHW cadre can be locally owned with significant community and local governance engagement, be well-integrated into existing primary health care systems, and have the necessary supportive supervision to optimize impact.

Scenario 1 decreases CHW salaries relative to the pilot program. From a human capital development perspective, this reduction is undesirable as higher salaries enable further opportunity and empower women CHWs, many of whom may be otherwise unemployed and/or less socioeconomically empowered. However, in the context of the health care system and current minimum wage standards, a lower salary may also increase feasibility and avoid potential perceptions of salary inequity. This could improve collaboration and integration of the cadre into the health care system. We believe this scenario is more feasible in the current political climate.

Scenarios 2 and 3 present important opportunities for further integration of CHWs into local primary health care systems, as well as improved ownership by local governance bodies and stakeholders. Strong linkages to primary health care systems and community engagement are key elements to CHW programmatic success and thus are included in the WHO guidelines for CHW program design,^4^ making this an important potential benefit of both scenarios. Conversely, the lack of dedicated supervisors in these scenarios poses risks to implementation of effective supportive supervision practices, training, monitoring and evaluation, and supply chain management, with scenario 3 posing greater risk in these regards.

In other examples of CHW program implementation, CHW supervisors who have additional responsibilities (e.g., providing clinical services at the local health post) have experienced challenges providing the supportive supervision that WHO guidelines recommend, including regular coaching and mentoring of CHWs, direct observation of CHW service delivery, and review of performance data and community feedback.^4^^,^^20^ These challenges may be for multiple contextually specific reasons. Health care facility staff who are asked to supervise CHWs, in addition to conducting their routine clinical or administrative responsibilities, frequently do not have the availability, training, or appropriately aligned incentives to optimize supportive supervision. This situation can manifest in supervisors infrequently visiting communities to observe CHW service delivery or having limited time and capacity to routinely review data, coach, mentor, and provide performance feedback. Scenarios 2 or 3 may pose similar risks.

As discussed previously, over the last 40 years, Nepal’s community health care system has included multiple community-based cadres, both full-time and part-time, paid and volunteer, including VHWs, community health leaders, MCHWs, ANMs, AHWs, and FCHVs.^8^^,^^12^^,^^18^^,^^40^^,^^41^ Now, with renewed and increasing enthusiasm to bolster progress toward the SDGs through community-based cadres, including growing recognition of the need to enhance capacity in the FCHV cadre, paired with the opportunity of increasing fiscal space at the federal and provincial levels, there is an important opportunity to offer guidance on how improvements in the community health care system can be optimally implemented.^17^

Because the pilot described here was designed before the increasingly popular concept of ANM- or AHW-based community services, it did not incorporate these cadres specifically into the pilot methodology. Notably, several CHWs employed in this pilot program were in fact ANMs. However, this was not an intentional aspect of the pilot program protocol, and performance of CHWs who had ANM qualifications were not compared to CHWs without ANM certification. As such, while it may ultimately be the case that AHWs and/or ANMs are well-positioned to carry forward the work of such a community-based cadre as described in this pilot, the data included in this manuscript cannot specifically comment on this question. Further research detailing the feasibility of the ANM or AHW cadre leveraged in this particular capacity should be considered to address these questions.

Finally, it is important to recognize the opportunity a ‘dual-cadre’ system provides in which paid full-time CHWs work closely with a volunteer cadre to optimize community-based service delivery. Dual-cadre systems are exemplified in other countries, including Ethiopia’s health extension workers and health development army volunteers. These systems have been employed historically in Nepal as well, with FCHVs working in collaboration with VHWs and MCHWs, and now in various capacities with ANMs and AHWs at health posts and primary health care centers.^12^ Similarly, the potential to further leverage the extensive reach, infrastructure, and effectiveness of the FCHV network with a new CHW cadre is significant. The CHWs in the pilot described here interact regularly with FCHVs, including attending FCHV meetings, health campaigns, and collaborating on health promotion activities. As CHWs are charged with enumerating and enrolling all members of every household in their respective catchment areas, they have also historically been accompanied by FCHVs during household visits. This has been helpful to ensure CHWs do not miss households or family members while conducting triage and referral care and community-based diagnosis, treatment, and counseling. Expansion of a full-time, paid community-based cadre could more deeply and effectively engage FCHVs (e.g., around outreach activities, civil registration and vital statistics, and routine monitoring and evaluation activities related to government reporting). Additionally, a CHW cadre like the one described in the pilot may further bolster supervision of FCHVs to include an enhanced focus on regular skill development, problem solving, performance review, professional development, and data feedback loops as part of routine work.

### Limitations

Our study includes several limitations. First, our analysis regarding CHW time allocation was conducted using top-down allocations of costs from CommCare. Practically, this equates time required for CHWs to complete a form on mobile phones using the CommCare application as a proxy for time spent providing care; however, this proxy has not been validated. During site visits, the use of Commcare tools were observed, and CHWs also provided self-reported average time required to conduct a specific type of home visit. No notable differences were found between time stamps from the CommCare tool and reported numbers. Nonetheless, differences here could impact our analysis. Additionally, some CHW services and functions (e.g., postpartum contraception counseling or time required for travel) are not accurately captured using this methodology; therefore, it is difficult to determine precise costs for these aspects of service delivery. However, given that the top-down costing approach includes all costs, these limitations should not impact overall per capita cost. More detailed analysis via time-driven activity-based costing would be more rigorous, but with human resource and financial constraints, this was not feasible.

Second, given that this analysis includes only 2 municipalities, the external validity of our conclusions to other areas throughout Nepal are potentially limited. The full pilot will cover over 250,000 persons across 2 districts. The analysis presented includes a subset catchment area of 60,504 persons in 1 district. Additionally, as noted, there was incomplete implementation of group ANC and NCD services during the measurement period, which may have underestimated per capita costs accordingly. Future analysis will include the full catchment area when all service delivery is implemented, but such analysis is not expected for at least 2 more years. Given the delay anticipated, these data provide some early insights that may help inform decision making in the current policy context.

Third, our analysis excluded costs for some administrative personnel involved in programmatic design. We do not believe such personnel would be scalable for the program as they are not involved in service delivery, and budgetary allocations are likely to be constrained if the government chooses to scale the program in other geographies. However, the exclusion of such personnel presumes limited further ongoing design and iteration which may cause challenges for the program’s operations. Additionally, these personnel do enhance oversight of the program currently and their absence could affect programmatic quality. Notably, such functions could also be fulfilled by local governance bodies or primary health care facility staff, as described in scenarios 2 and 3.

Finally, the pilot was implemented in the context of a public-private partnership, through which Nyaya Health Nepal oversees day-to-day operations of the public-sector Bayalpata Hospital; therefore, the generalizability of these results for other public-sector institutions should be interpreted with caution. Future scale of a CHW cadre is more likely in public-sector or strictly private-sector settings, as public-private partnerships remain limited in Nepal. This may have overestimated costs of early-stage program development as it includes a higher administrative staffing ratio than would be necessary in future at-scale efforts. Scenarios 1, 2, and 3 attempt to account for this discrepancy by adjusting to the expected minimum wage standard (scenario 1), and assuming public-sector staffing for administrative functions (scenarios 2 and 3). Conversely, our analysis does not include potential costs for administrative personnel overseeing scaling efforts if this program were to be adopted more broadly. Similarly, the unique management structure of the pilot is not generalizable, and this may impact feasibility of a similar program in different contexts. Additionally, the catchment area also includes a hospital run by the same public-private partnership that offers access and quality of services not necessarily generalizable to other regions. Accessible, high-quality, facility-based services are a strong determinant of CHW program impact,^4^ thus similar results in areas with less access to quality health care facilities may be less feasible.

## CONCLUSION

As Nepal looks ahead toward achieving UHC and SDG targets, more robust primary health systems are required. A new CHW cadre, such as assessed in this national pilot, represents an important opportunity. The costs described may be instructive for policy makers and locally elected officials in Nepal and may also be relevant to countries with similar health care settings aiming to improve community health care systems on their path toward the SDGs.

## Supplementary Material

19-00393-Schwarz-Supplement_2.pdf

19-00393-Schwarz-Supplement_1.pdf

19-00393-Schwarz-Supplement_3.pdf

## Notes

**Peer Reviewed**

**Funding:** This work was supported by the United States Agency for International Development (USAID) via a Partnerships for Enhanced Engagement in Research award [sponsor grant number AID-OAA-A-11-00012 and a National Academy of Science subaward letter 2000007780] and by the Office of the Director, National Institutes of Health under an Early Independence Award [DP5OD019894] to Duncan Maru (the Eunice Kennedy Shriver National Institute of Child Health & Human Development (NICHD) and the National Institute of Dental & Craniofacial Research (NIDCR) provided support for this award). The funders played no role in research design, data collection, data analysis, manuscript write-up, or decision to publish. Any opinions, findings, conclusions, or recommendations expressed in this article are those of the authors alone and do not necessarily reflect the views of the USAID or the National Academy of Science.
