# Supplementary material for: Costing Analysis of a Pilot Community Health Worker Program in Rural Nepal
Source: Glob Health Sci Pract. 2020 Jun 30;8(2):239–55. doi: 10.9745/GHSP-D-19-00393 (PMC7326517; doi:10.9745/GHSP-D-19-00393)
Supplement: 19-00393-Schwarz-Supplement_2.pdf [file 19-00393-Schwarz-Supplement_2.pdf]

Supplement to: Nepal P, Schwarz R, Citrin D, et al. Costing analysis of a pilot community health worker program in rural Nepal. *Glob Health Sci Pract*. 2020;8(2). https://doi.org/10.9745/GHSP-D-19-00393

Supplementary File 2. Costing of Community Health Worker Pilot Program and Alternative Implementation Scenarios

Description	CHW Pilot (Actual)			Scenario 1			Scenario 2			Scenario 3		
	Sanfebagar	Kamalbazaar	Total	Sanfebagar	Kamalbazaar	Total	Sanfebagar	Kamalbazaar	Total	Sanfebagar	Kamalbazaar	Total
Part A: Disaggregation of Total Costs												
Personnel Costs (A)	$92,264	$43,796	$136,060	$61,589	$34,899	$96,488	$50,520	$29,225	$79,746	$34,758	$20,123	$54,881
Compensation of CHWs	$65,433	$29,020	$94,453	$34,758	$20,123	$54,881	$34,758	$20,123	$54,881	$34,758	$20,123	$54,881
Compensation of CHNs	$15,762	$9,102	$24,864	$15,762	$9,102	$24,865	$15,762	$9,102	$24,865	-	-	-
Compensation of CHPAs	$11,069	$5,674	$16,743	$11,069	$5,674	$16,742	-	-	-	-	-	-
Other Direct Expenses (B)	$6,531	$6,656	$13,187	$6,530	$6,655	$13,186	$6,530	$6,655	$13,186	$1,765	$1,020	$2,785
Medical consumables, lab reagents, etc.	$5,793	$5,918	$11,711	$5,793	$5,918	$11,710	$5,793	$5,918	$11,710	$1,765	$1,020	$2,785
Depreciation of ultrasound equipment	$738	$738	$1,476	$738	$738	$1,475	$738	$738	$1,475	-	-	-
Indirect Expenses (C)	$19,532	$15,726	$35,258	$19,533	$15,727	$35,259	$4,803	$4,228	$9,031	$4,070	$3,394	$7,464
Staff benefits and uniform	$2,693	$2,919	$5,612	$2,693	$2,919	$5,612	$602	$421	$1,024	$600	$421	$1,022
Jeep rental expenses	$11,699	$8,742	$20,441	$11,699	$8,742	$20,441	-	-	-	-	-	-
Regular supplies and administrative	$2,198	$2,467	$4,665	$2,198	$2,467	$4,666	$2,198	$2,434	$4,632	$2,198	$2,052	$4,250
Digital tools and network expenses	$2,942	$1,598	$4,540	$2,942	$1,598	$4,540	$2,003	$1,372	$3,375	$1,272	$921	$2,192
TOTAL COSTS (A+B+C)	$118,327	$66,178	$184,505	$87,652	$57,281	$144,933	$61,854	$40,108	$101,962	$40,593	$24,537	$65,130
Part B: Disaggregation of Administrative Functions												
Total cost of Administrative Functions	$49,651	$27,034	$76,685	$44,853	$25,182	$70,036	$23,546	$14,805	$38,350	$6,091	$4,905	$10,996
Program planning, procurement & admin	$11,355	$6,137	$17,492	$10,397	$5,859	$16,257	$3,941	$2,391	$6,332	$1,212	$734	$1,946
Continuous education & training	$8,689	$4,575	$13,264	$6,693	$3,996	$10,690	$4,765	$2,907	$7,672	$2,526	$1,530	$4,055
Supervision, monitoring, and evaluation	$21,837	$10,466	$32,303	$21,837	$10,466	$32,302	$10,258	$5,524	$15,782	-	-	-

Supplement to: Nepal P, Schwarz R, Citrin D, et al. Costing analysis of a pilot community health worker program in rural Nepal. *Glob Health Sci Pract*. 2020;8(2). https://doi.org/10.9745/GHSP-D-19-00393

**Description**	**CHW Pilot (Actual)**			**Scenario 1**			**Scenario 2**			**Scenario 3**		
	**Sanfebagar**	**Kamalbazaar**	**Total**	**Sanfebagar**	**Kamalbazaar**	**Total**	**Sanfebagar**	**Kamalbazaar**	**Total**	**Sanfebagar**	**Kamalbazaar**	**Total**
Data learning and reporting	$5,749	$3,123	$8,872	$4,792	$2,846	$7,637	$3,510	$2,132	$5,642	$1,212	$734	$1,946
Continuous surveillance	$2,021	$2,733	$4,754	$1,134	$2,016	$3,150	$1,072	$1,850	$2,922	$1,141	$1,907	$3,048
Part C: Cost Per Capita by Programs												
Total cost per capita	$3.22	$2.80	$3.05	$2.38	$2.41	$2.40	$1.68	$1.69	$1.69	$1.10	$1.03	$1.08
Pregnancy surveillance system	$0.76	$0.72	$0.75	$0.55	$0.61	$0.57	$0.39	$0.45	$0.41	$0.29	$0.35	$0.31
Antenatal care	$0.34	$0.62	$0.45	$0.24	$0.52	$0.35	$0.17	$0.38	$0.25	$0.12	$0.28	$0.19
Group antenatal care	$0.32	$0.59	$0.42	$0.29	$0.57	$0.40	$0.19	$0.33	$0.25	-	-	-
Postnatal care	$0.26	$0.41	$0.32	$0.19	$0.34	$0.25	$0.14	$0.25	$0.18	$0.11	$0.19	$0.14
Under 2 registry and malnutrition	$0.57	$0.45	$0.52	$0.41	$0.37	$0.40	$0.29	$0.28	$0.29	$0.22	$0.21	$0.22
Chronic disease management	$0.99	-	$0.59	$0.70	-	$0.43	$0.50	-	$0.30	$0.36	-	$0.22
Part D: Cost Per Beneficiary by Programs												
Pregnancy surveillance system	$6.58	$4.75	$5.74	$4.76	$3.99	$4.41	$3.39	$2.97	$3.20	$2.53	$2.29	$2.42
Antenatal care	$12.26	$13.47	$12.89	$8.85	$11.25	$10.10	$6.27	$8.27	$7.31	$4.64	$6.15	$5.42
Group antenatal care	$29.31	$25.43	$27.06	$26.67	$24.62	$25.48	$17.97	$14.13	$15.74	-	-	-
Postnatal care	$5.40	$4.95	$5.16	$3.96	$4.13	$4.05	$2.88	$3.04	$2.96	$2.22	$2.31	$2.27
Under 2 registry and malnutrition	$12.72	$10.59	$11.91	$9.21	$8.88	$9.09	$6.57	$6.58	$6.57	$4.91	$4.99	$4.94
Chronic disease management	$20.18	-	$20.18	$14.49	-	$14.49	$10.19	-	$10.19	$7.40	-	$7.40

Abbreviations: CHN, community health nurse; CHPA, community health program associate, CHW, community health worker
