# Supplementary material for: Costing Analysis of a Pilot Community Health Worker Program in Rural Nepal
Source: Glob Health Sci Pract. 2020 Jun 30;8(2):239–55. doi: 10.9745/GHSP-D-19-00393 (PMC7326517; doi:10.9745/GHSP-D-19-00393)
Supplement: 19-00393-Schwarz-Supplement_1.pdf [file 19-00393-Schwarz-Supplement_1.pdf]

Supplement to: Nepal P, Schwarz R, Citrin D, et al. Costing analysis of a pilot community health worker program in rural Nepal. *Glob Health Sci Pract*. 2020;8(2). https://doi.org/10.9745/GHSP-D-19-00393

Supplementary File 1. Costing Methods and Definitions Used in Community Health Worker Pilot Program, Nepal

Programmatic Information	Description	Rationale	Data Source
Population and number of households	Population and number of households of Sanfebagar and Kamalbazaar disaggregated by wards	To identify costs per capita of delivering the program at the municipal and ward level	National census 2011 and data available from continuous surveillance
Number of beneficiaries	Number of unique individuals who were provided direct care by a CHW in the given period	To identify costs per beneficiary of all individual programs at municipal and ward level	*CommCare* data collected by CHWs during routine care
Number of encounters	Number of encounters by a CHW for one of the programs or household surveillance	To identify the time spent by CHWs on individual programs and to calculate capacity costs	The program notes and forms filled out by CHWs in *CommCare*
Number of events	Number of hub meetings, training days and supervision field visits	To identify events relating to design elements and estimate time spent on specific events	Review of program workflows, calendars, and staff operations
**Cost Description**	**Description**	**Allocation Metric**	**Data Source**
Compensation of CHWs	Total annual compensation inclusive of base salary, allowances, retirement benefits	CHW time spent on programs and proportionate allocation of remainder time across programs	Average time required to fill out specific forms on *CommCare* for each CHW
Compensation of CHNs and CHPA/s	Total annual compensation inclusive of base salary, allowances, retirement benefits, external trainings	CHN and CHPA time spent on program supervision as well as other design elements	Review of workflows, calendars, and financial details via financial records
Other direct expenses	Medical consumables, lab reagents and depreciation of medical equipment	Actual usage of consumables and lab reagents; depreciation of equipment for 5 years of useful life	Consumables, reagents and equipment allocations as recorded in inventory management platform and financial records
Indirect expenses	Staff benefits, jeep rentals, depreciation of digital tools, network expenses and other admin expenses	Number of field visits related to care delivery; allocation by personnel for benefits and digital tools	Via financial records
**Administrative Function**	**Description**	**Allocation Metric**	**Data Source**
Planning and administration	Costs related to building program calendars, managing logistics, and stakeholder engagement	Intermediate allocations by CHW, CHN and CHPA time allocation, final allocation by CHW encounters	Review of program workflows, calendars, and staff operations
Training	Costs related to trainings during hub meetings as well as quarterly programmatic trainings	Intermediate allocations by CHW, CHN and CHPA time allocation, final allocation by CHW encounters	Review of program workflows, calendars, and staff operations
Supervision, monitoring, and evaluation	Costs related to on the job mentoring, supervision field visits and 1:1 coaching	Intermediate allocations by CHW, CHN and CHPA time allocation, final allocation by CHW encounters	Review of program workflows, calendars, and staff operations

Supplement to: Nepal P, Schwarz R, Citrin D, et al. Costing analysis of a pilot community health worker program in rural Nepal. *Glob Health Sci Pract*. 2020;8(2). https://doi.org/10.9745/GHSP-D-19-00393

**Programmatic Information**	**Description**	**Rationale**	**Data Source**
Data reporting and learning	Costs related to usage of digital tools and data learning sessions at hub meetings	Intermediate allocations by CHW, CHN and CHPA time allocation, final allocation by CHW encounters	Review of program workflows and staff operations
Continuous surveillance	Costs relating to updating of household and demographic information during routine care	CHW time spent on updating demographic and household details, final allocation by encounters	Average time required to fill out specific forms on *CommCare* for each CHW

Abbreviations: CHN, community health nurse; CHPA, community health program associate, CHW, community health worker.
