# Supplementary material for: Costing Analysis of a Pilot Community Health Worker Program in Rural Nepal
Source: Glob Health Sci Pract. 2020 Jun 30;8(2):239–55. doi: 10.9745/GHSP-D-19-00393 (PMC7326517; doi:10.9745/GHSP-D-19-00393)
Supplement: 19-00393-Schwarz-Supplement_3.pdf [file 19-00393-Schwarz-Supplement_3.pdf]

Supplement to: Nepal P, Schwarz R, Citrin D, et al. Costing analysis of a pilot community health worker program in rural Nepal. *Glob Health Sci Pract*. 2020;8(2). https://doi.org/10.9745/GHSP-D-19-00393

Supplementary File 3. Nepal Health Care Expenditure Context and Targets

Description	2015/16	Source	2030	Source
Per capita gross domestic product, USD$	759	IMF Country Report, 2018	2,500	Nepal SDG Goals - Status and Roadmap 2016-30
Total population, in millions	28	Nepal Population Report, 2016	32.4	Nepal Population Report, 2016
Total gross domestic product, USD$ million	21,254	IMF Country Report, 2018	81,000	Nepal SDG Goals - Status and Roadmap 2016-30
GDP in health care expenditure, %	6.72	Nepal National Health Accounts 2012/13-2015/16	7.00	Nepal SDG Goals - Status and Roadmap 2016-30
Per capita health care expenditure, USD$	51	Nepal National Health Accounts 2012/13-2015/16	175	Nepal SDG Goals - Status and Roadmap 2016-30
Government spending in health care expenditure, %	27	Nepal National Health Accounts 2012/13-2015/16	44	Nepal SDG Goals - Status and Roadmap 2016-30
Total government health care expenditure, USD$ million	13.60	Nepal National Health Accounts 2012/13-2015/16	77.00	Nepal SDG Goals - Status and Roadmap 2016-30
Out-of-pocket expenditure, %	52	Nepal National Health Accounts 2012/13-2015/16	35	Nepal SDG Goals - Status and Roadmap 2016-30
Other sources, %	21	Nepal National Health Accounts 2012/13-2015/16	21	Nepal SDG Goals - Status and Roadmap 2016-30

Abbreviations: GDP, gross domestic product; IMF, International Monetary Fund; SDG, Sustainable Development Goal.

References

International Monetary Fund. *Nepal: 2018 Article IV Consultation—Press Release; Staff Report; And Statement by the Executive Director for Nepal*. Washington, DC: International Monetary Fund; 2019.

Population Education & Health Research Center Ltd. *Nepal Population Report.* Kathmandu, Nepal: Population Education & Health Research Center; 2016.

Ministry of Health and Population. *Nepal National Health Accounts 2012/13 to 2015/16*. Kathmandu, Nepal: Ministry of Health and Population; 2018.

United Nations Development Program. *Sustainable Development Goals Status and Roadmap: 2016-2030, Nepal*. Kathmandu, Nepal: Government of Nepal, National Planning Commission; 2017.
